# What is the ideal dose and power output of low-level laser therapy (810 nm) on muscle performance and post-exercise recovery? Study protocol for a double-blind, randomized, placebo-controlled trial

**DOI:** 10.1186/1745-6215-15-69

**Published:** 2014-02-27

**Authors:** Adriano Rodrigues de Oliveira, Adriane Aver Vanin, Thiago De Marchi, Fernanda Colella Antonialli, Vanessa dos Santos Grandinetti, Paulo Roberto Vicente de Paiva, Gianna Móes Albuquerque Pontes, Larissa Aline Santos, Ivo de Oliveira Aleixo Junior, Paulo de Tarso Camillo de Carvalho, Jan Magnus Bjordal, Ernesto Cesar Pinto Leal-Junior

**Affiliations:** 1 Postgraduate Program in Biophotonics Applied to Health Sciences, Universidade Nove de Julho (UNINOVE), Rua Vergueiro 235, São Paulo, SP 01504-001, Brazil; 2 Postgraduate Program in Rehabilitation Sciences, Universidade Nove de Julho (UNINOVE), Rua Vergueiro 235, São Paulo, SP 01504-001, Brazil; 3 Sports Medicine Institute, University of Caxias do Sul, Rua Francisco Getulio Vargas 1130, Caxias do Sul, RS 95070-560, Brazil; 4 Physiotherapy Research Group, Faculty of Medicine and Dentistry, University of Bergen, Kalfarveien 31, Bergen 5020, Norway

**Keywords:** Low-level laser therapy, Skeletal muscle fatigue, Skeletal muscle recovery, Phototherapy parameters, Exercise

## Abstract

**Background:**

Recent studies involving phototherapy applied prior to exercise have demonstrated positive results regarding the attenuation of muscle fatigue and the expression of biochemical markers associated with recovery. However, a number of factors remain unknown, such as the ideal dose and application parameters, mechanisms of action and long-term effects on muscle recovery. The aims of the proposed project are to evaluate the long-term effects of low-level laser therapy on post-exercise musculoskeletal recovery and identify the best dose andapplication power/irradiation time.

**Design and methods:**

A double-blind, randomized, placebo-controlled clinical trial with be conducted. After fulfilling the eligibility criteria, 28 high-performance athletes will be allocated to four groups of seven volunteers each. In phase 1, the laser power will be 200 mW and different doses will be tested: Group A (2 J), Group B (6 J), Group C (10 J) and Group D (0 J). In phase 2, the best dose obtained in phase 1 will be used with the same distribution of the volunteers, but with different powers: Group A (100 mW), Group B (200 mW), Group C (400 mW) and Group D (0 mW). The isokinetic test will be performed based on maximum voluntary contraction prior to the application of the laser and after the eccentric contraction protocol, which will also be performed using the isokinetic dynamometer. The following variables related to physical performance will be analyzed: peak torque/maximum voluntary contraction, delayed onset muscle soreness (algometer), biochemical markers of muscle damage, inflammation and oxidative stress.

**Discussion:**

Our intention, is to determine optimal laser therapy application parameters capable of slowing down the physiological muscle fatigue process, reducing injuries or micro-injuries in skeletal muscle stemming from physical exertion and accelerating post-exercise muscle recovery. We believe that, unlike drug therapy, LLLT has a biphasic dose–response pattern.

**Trial registration:**

The protocol for this study is registered with the Protocol Registry System, ClinicalTrials.gov identifier NCT01844271.

## Background

Muscle fatigue is a dynamic, time-dependent failure to maintain a desired level of yield or work during a repetitive or sustained activity [1]. Fatigue has a central and peripheral component, both of which affect the production of force during muscle performance. The decline in muscle activity varies in accordance with the type of muscle fiber and intensity of the exercise [2,3]. Fatigue can also contribute to the development of muscle damage, which is characterized by an inflammatory response [4,5], an increase in muscle proteins in the blood [6] and late-onset muscle pain [7-9]. These events occur in a more accentuated fashion when eccentric contractions are employed [6,10,11].

High-performance athletes are prone to muscle damage stemming from training and competition, compromising their performance for a given period of time, which can range from minutes or hours to several days or even months [12]. A large number of therapies are currently employed after sport activities to enhance muscle recovery, such as active recovery [13-15], cryotherapy [9,16], stretching [12] and electrical stimulation [17]. The aim of post-exercise therapy is to reverse muscle damage, with no attempt made to prevent or attenuate the occurrence of muscle damage.

Recent studies by our research group involving low-level laser therapy (LLLT) and LED therapy have demonstrated positive results when applied prior to exercise, with the attenuation of muscle fatigue [18-24] and favoring the recovery of biochemical markers related to muscle damage [25-27]. The findings suggest that LLLT can minimize the occurrence of injury and optimize an athlete’s return to his/her activities in a shorter period of time.

Despite the positive results obtained in the studies cited above, a number of variables remain unknown, such as the ideal dose and application parameters, mechanisms of action and long-term effects on muscle recovery. Based on our line of research, LLLT is a valuable tool for the attenuation of muscle fatigue and the acceleration of post-exercise muscle recovery. As the same biphasic dose–response pattern has been seen in other musculoskeletal disorders and both power and irradiation time exert an influence on the efficacy of therapy, these outcomes justify the development of the proposed study.

## Methods and study design

### Characterization and purpose of study

A double-blind, placebo-controlled, randomized, clinical trial will be carried out in two phases. The study will be conducted at the Laboratory of Phototherapy in Sport and Exercise at Nove de Julho University (UNINOVE) in the city of São Paulo, Brazil. The project has received approval from the Human Research Ethics Committee of the university under protocol number 397774/2011 and the study is supported by a Young Researcher award grant from the Brazilian fostering agency, Sao Paulo Research Foundation (FAPESP), under process number 2010/52404-0.

### Characterization of sample

Twenty-eight male high-level soccer athletes from the same team will participate in each phase of the study. The decision to recruit volunteers from the same team was made to enhance the homogeneity of the sample. For recruitment of volunteers an initial contact will be made with head coaches of several Brazilian soccer teams. If the head coach accepts that the team can be part of the study, then a personal invitation will be made for athletes individually. Afterwards, the research team will ask athletes about their personal data in order to identify if they meet the inclusion criteria or if they need to be excluded from the clinical trial.

### Calculation of sample size

The sample size was calculated based on a previous study [28], in which a similar experimental model and exercise protocol was employed as that to be used in the proposed study. The sample size calculation considered a β of 20% and α of 5%. In the study used as reference for this calculation of Baroni *et al*. [28], LLLT led to the post-exercise recovery of creatine kinase (CK) (muscle injury marker) to 435.95 U/l (standard deviation: 238.04), whereas placebo treatment led to an increase in CK to 1,327.58 ± 949.82 U/l. Using these parameters, a total of seven volunteers would be needed for each of the four groups in each phase of the study (total of 28 volunteers per phase).

### Inclusion criteria

The following inclusion criteria will be employed:

•Professional soccer athletes;

•Age between 18 and 35 years;

•Male gender;

•Minimum of 80% participation in team practice sessions;

•Light or intermediate skin color [29];

•Agreement to participate through signed statement of informed consent.

### Exclusion criteria

Participants with the following will be excluded from the study:

•History of musculoskeletal injury to hips or knees in the previous 2 months;

•Use of pharmacological agents or nutritional supplements;

•Occurrence of musculoskeletal injury during the study;

•Any change in practice routine in relation to the rest of the team during the study.

### Composition of groups and randomization process

**Figure 1:**
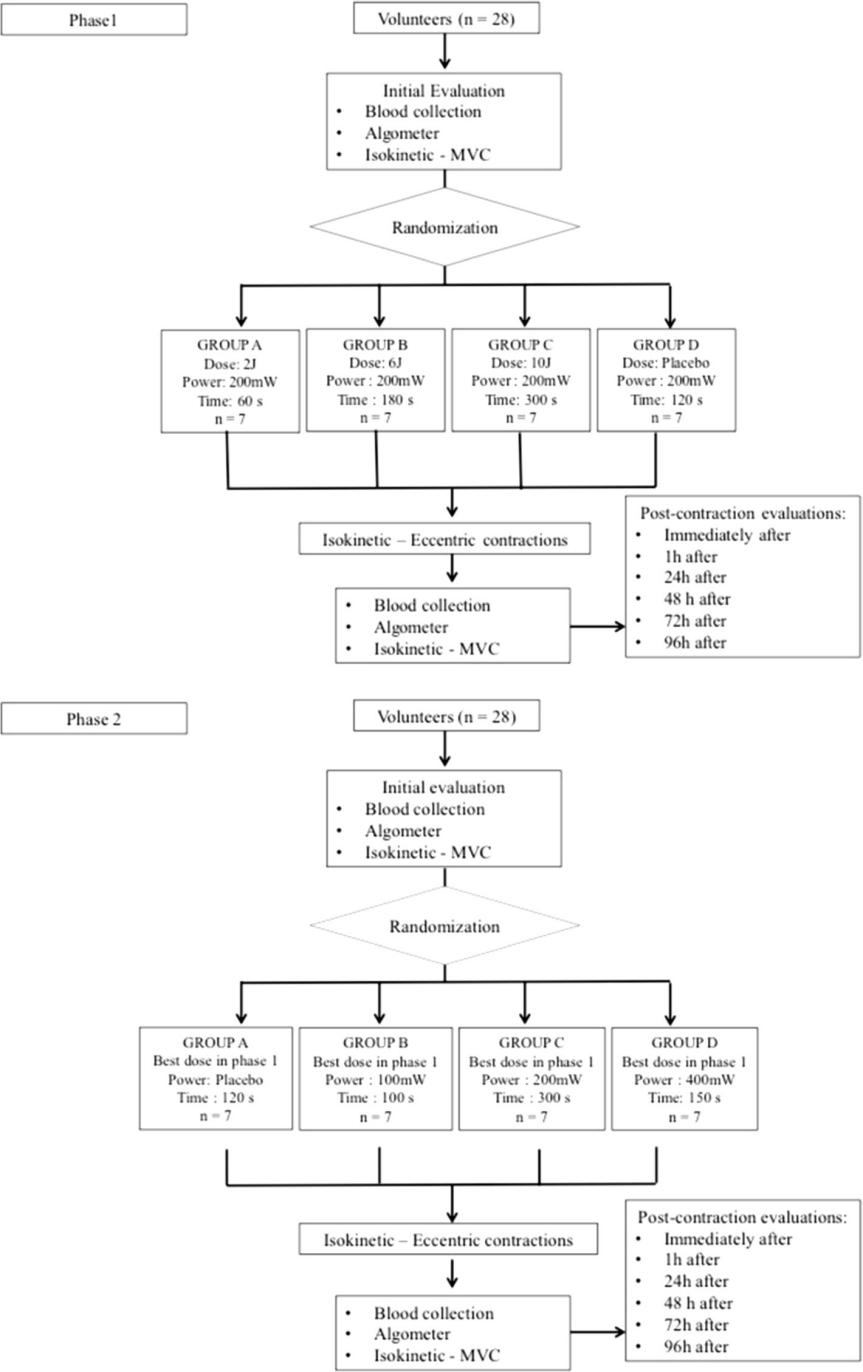
**Flowchart of design**, **composition of groups and protocols in phases 1 and 2.** MVC, maximum voluntary contraction.

The volunteers will be randomly allocated to four experimental groups (n = 7 per group) based on the LLLT dose (phase 1) and power (phase 2) (Figure 1). Randomization will be carried out by a simple drawing of lots (A, B, C or D). The laser unit will emit the same sound regardless of the programmed power output. Randomization labels will be created using a randomization table at a central office, where a series of sealed, opaque, numbered envelopes will be used to ensure confidentiality. Randomization will be conducted by a participating researcher, who will have the function of programming the laser device based on the randomization results. This researcher will be instructed not to inform the participants or other researchers regarding the LLLT dose (phase 1) or power (phase 2) until the study has been completed. Thus, the researcher in charge of the administration of the LLLT will be blinded to the dose and power applied to the volunteers.

### Experimental protocol

#### Evaluations and informative procedures

Evaluations will be carried out before and at the end of the isokinetic protocol by a researcher blinded to the LLLT dose (phase 1) and power (phase 2). The volunteers will then be informed about the procedures and will sign a statement of informed consent in compliance with Resolution 196/96 of the Brazilian National Board of Health prior to the execution of the study. All individuals in both phases will be submitted to the same evaluation protocol, which will involve the aspects described below.

#### Personal data

A questionnaire will be administered addressing age (years), body mass (kilograms), height (centimeters), dominant lower leg, schooling (no schooling, elementary school education, high school education, university education or postgraduate degree) and marital status (single, married or widowed).

#### Blood samples and biochemical analyses

Following the informative process and randomization, blood samples (10 ml) will be collected from the antecubital vein before and 1 minute after the eccentric contraction protocol. Blood samples will also be collected 1, 24, 48, 72 and 96 hours after the protocol. The samples will be taken by a nurse blinded to the allocation of the volunteers to the four experimental groups. One hour after collection, each sample will be centrifuged at 3,000 rpm for 20 minutes. Pipettes will be used to transfer the serum to Eppendorf® tubes (Eppendorf, Hamburg, Germany), which will be stored at −80°C until analysis.

Blood analysis will involve the determination of CK and lactate dehydrogenase (LDH) activity as indirect markers of muscle damage, using spectrophotometry and specific reagent kits (Labtest Diagnóstica, Lagoa Santa, MG, Brazil); IL-1β, IL-6 and TNF-α levels as inflammatory markers, using ELISA and specific reagents (R&D Systems, Minneapolis, MN, USA); and thiobarbituric acid reactive substances (TBARS), carbonylated proteins, catalase (CAT) and superoxide dismutase (SOD) as markers of oxidative stress, using spectrophotometry and specific reactions.

#### Evaluation of delayed onset muscle soreness (DOMS)

Delayed onset muscle soreness (DOMS) will be evaluated based on the pressure pain threshold, using an analog algometer (Baseline®, Rome, Italy). This device consists of a rod with a rounded rubber tip coupled to a pressure (force) meter. The display presents values in kilograms. As the surface of the rubber tip measures 1 cm^2^, the reading is expressed in kilograms per square centimeter (kg/cm^2^). Values range from 0 to 10 kg, with a precision of 0.1 kg. The most sensitive areas of the knee extensors (medial, lateral and central) of the non-dominant lower limb will be located through palpation by an examiner blinded to the allocation of the volunteers to the different groups and will be marked with a dermographic marker. The cylindrical end of the equipment will be positioned perpendicularly to the demarcated area. Pressure will be applied to the surface of the skin with a gradual increase in increments of 0.1 kg. The volunteers will be instructed to say ‘yes’ when the pressure exerted becomes painful. Three measures will be taken with the algometer on the same demarcated point of the aforementioned muscle sites. The mean pressure pain threshold will be determined from the three readings of each of the three sites and the mean values will be used in the statistical analysis. Readings will be taken prior to stretching and warm-up, 1 minute after the eccentric contraction protocol as well as 1, 24, 48, 72 and 96 hours after the execution of the protocol.

#### Stretching and warm-up

Prior to the isokinetic protocol, the volunteers will perform three 60-second sets of active stretching of the knee extensors of the non-dominant lower limb. The volunteers will then perform a warm-up exercise consisting of pedaling a stationary bike (Inbramed, Porto Alegre, RS, Brazil) at 100 rpm for 5 minutes without load.

#### Isokinetic protocol: maximum voluntary contraction (MVC)

An isometric dynamometer will be used for the evaluation of muscle function and the execution of the exercise protocol. This instrument is considered the method with the greatest reliability for the measure of the musculoskeletal performance. Immediately after the stretching and warm-up exercises, the maximum voluntary contraction (MVC) test will be performed. For such, the volunteers will sit on the seat of the isokinetic dynamometer (System 4, Biodex Medical Systems, Inc., Shirley, NY, USA) with an angle of 100° between the trunk and hip and the non-dominant leg positioned with the knee at 60° of flexion (0° corresponds to complete knee extension) and attached to the seat of the dynamometer by a strap. The dominant leg will be positioned at 100° of hip flexion and will also be attached to the seat by a strap. The volunteers will also be attached to the seat of the dynamometer through the use of two straps crossing the trunk. The volunteers will be instructed to cross their arms over the trunk and the axis of the dynamometer will be positioned parallel to the center of the knee. The MVC test will consist of three 5-second isometric contractions of the knee extensors of the non-dominant leg. The highest torque value of the three contractions (peak torque) will be used for the statistical analysis. The choice of this parameter is due to the fact that this variable reflects the maximum generation of force by the muscle. Instructions on how to execute the test will be given first and the volunteers will receive verbal encouragement during the execution of the test. This test has demonstrated reliability and reproducibility in a previous study carried out [28]. The MVC will be performed immediately (1 minute) after the eccentric contraction protocol as well as 1, 24, 48, 72 and 96 hours after the eccentric contraction protocol to evaluate post-exercise muscle recovery.

### Low-level laser therapy (LLLT)

#### Specifications

A five-diode cluster laser device (each cluster with 0.0364 cm^2^; wavelength: 810 nm; power: 0, 100, 200 or 400 mW in each diode; continuous light emission) (Thor Photomedicine, Chesham, UK) will be used for LLLT. The functioning of the laser will be verified prior to the treatment of each volunteer, with an inspection of the energy source, application points and energy measurement. To ensure blinding, the device will emit the same sounds regardless of the programmed mode (active or placebo).

#### Variation of dose (phase 1)

In phase 1, the output power will be fixed at 200 mW and LLLT will be applied 2 minutes prior to the pre-exercise MVC test with the cluster in direct contact with the skin at six distinct sites of the knee extensor musculature of the non-dominant limb (two medial, two lateral and two central sites). As the cluster has five diodes and six different sites will be irradiated, a total of 30 points will be irradiated in the musculature. Based on the results of the randomization, the volunteers of the four experimental groups will receive the following doses: Group A, 2 J per diode, 10 J on each application of the cluster with 200 mW (10 seconds of irradiation at each site), 60 J of total irradiated energy on the muscle (60 seconds of total irradiation time); Group B, 6 J per diode, 30 J on each application of the cluster with 200 mW (30 seconds of irradiation at each site), 180 J of total irradiated energy on the muscle (180 seconds of total irradiation time); Group C, 10 J per diode, 50 J on each application of the cluster with 200 mW (50 seconds of irradiation at each site), 300 J of total irradiated energy on the muscle (300 seconds of total irradiation time); and Group D, 0 J per diode (placebo) (20 seconds of irradiation at each site, 120 seconds of total time, but without effective irradiation).

#### Variation of power (phase 2)

The aim of phase 2 is to determine the best power/irradiation time (Watts/s) for use on humans to enhance muscle recovery following physical exercise. For such, the dose with the best results in phase 1 will be used and the same methodological and evaluative measures will be employed. Thus, only the power of the equipment (and consequently irradiation time) will be varied (100, 200, 400 or 0 mW). As energy (E) expressed in Joules is equal to power (P) expressed in Watts multiplied by time (t) measured in seconds, different power variations will lead to the same total energy delivered to the tissue. *A priori*, if the best dose were determined to be 10 J, the following would be the parameters:

•10 J = 0.1 W (100 mW) × 100 s

•10 J = 0.2 W (200 mW) × 50 s

•10 J = 0.4 W (400 mW) × 25 s

•10 J = 0.0 W (placebo, 60 s)

### Isokinetic protocol: eccentric contractions

Precisely 3 minutes after the end of LLLT, the volunteers will perform the eccentric contraction protocol, which will consist of 75 eccentric isokinetic contractions of the knee extensor musculature in the non-dominant leg (five sets of 15 repetitions, 30-second rest interval between sets) at a velocity of 60°.seg^-1^ in both the eccentric and concentric movements with a 60° range of motion (between 90° and 30° of knee flexion). At each contraction, the dynamometer will automatically (passively) position the knee at 30°; the dynamometer will then flex the knee until reaching 90°. The volunteers will be instructed to resist the knee flexion movement imposed by the dynamometer with maximum force. Instructions on how to execute the maneuver will be given first and the volunteers will receive verbal encouragement throughout the protocol. Despite the diversity of protocols proposed for the execution of eccentric exercises on isokinetic dynamometers, the protocol described here was chosen based on a previous study carried out [28] in which this method proved effective and reproducible for the exercise-induced muscle damage.

### Data analysis

The intention-to-treat analysis will be followed. The primary outcome will be peak torque obtained from MVC at different time-points. Secondary outcomes will be biochemical markers CK, LDH, IL-1β, IL-6, TNF-α, TBARS, carbonylated proteins, CAT and SOD.

Data will be expressed as mean and standard deviation and will be firstly tested regarding normal distribution using Shapiro–Wilk test. ANOVA test with repeated measurements for the time factor will be performed to test between-groups differences (followed by the Bonferroni post hoc test). The level of significance for the statistical analysis will be set at 5% (*P* ≤0.05).

## Discussion

In the proposed study, the ideal LLLT application dose and power will be identified and the long-term effects of LLLT on post-exercise muscle fatigue and musculoskeletal recovery will be evaluated. For such, evaluations will be carried out of peak torque during MVC, late-onset muscle pain will be determined and biochemical analyses will be performed to determine changes in markers of inflammation, oxidative stress and muscle damage. The inflammatory process is known to be a defensive reaction against injury. This process has been extensively studied in its different aspects. Briefly, the main inflammation events are: 1) tissue injury; 2) the release of vasoactive substances by the injured tissue; 3) vasodilatation; 4) leukocyte adherence; 5) the migration of leukocytes from the blood stream to the injury site; and 6) tissue repair. Thus, muscle inflammation is a natural consequence of exercise-induced muscle damage [30].

Over time, LLLT exhibits a biphasic, dose–response pattern. This means that intermediate doses within a ‘therapeutic window’ trigger stimulation effects in biological tissue, whereas doses outside this range do not trigger any effects [31-34]. However, the therapeutic window varies depending on the condition to be treated [31]. Likewise, the power and irradiation time employed are extremely important to obtaining the best results. Thus, a short irradiation time and an output power that is either too low or too high can lead to null results [31,34]. Our intention, therefore, is to determine ideal laser therapy application parameters capable of slowing down the physiological muscle fatigue process, reducing injuries or micro-injuries in skeletal muscle stemming from physical exertion and accelerating post-exercise muscle recovery. We believe that, unlike drug therapy, LLLT has a biphasic dose–response pattern.

## Trial status

At the time of manuscript submission, the volunteers were being recruited.

## Abbreviations

ANOVA: Analysis of variance; CAT: Catalase; CK: Creatine kinase; DOMS: Delayed onset muscle soreness; ELISA: Enzyme-linked immunosorbent assay; FAPESP: Sao Paulo Research Foundation; IL: Interleukin; LDH: Lactate dehydrogenase; LED: Light-emitting diode; LLLT: Low-level laser therapy; MVC: Maximum voluntary contraction; SOD: Superoxide dismutase; TBARS: Thiobarbituric acid reactive substances; TNF: Tumor necrosis factor; UNINOVE: Nove de Julho University.

## Competing interests

ECPLJ receives research support from Multi Radiance Medical (Solon, OH, USA), a laser device manufacturer. Multi Radiance Medical had no role in the planning of this experiment, and the laser device used was not theirs. They had no influence on study design, decision to publish or preparation of the manuscript. The remaining authors declare that they have no conflicts of interest.

## Authors’ contributions

AAV, TDM, FCA and ECPLJ contributed to the conception and study design. ARO, VSG, PRVP, GMAP, LAS, IOAJ, PTCC, JMB and ECPLJ established the hypothesis and drafted the original proposal. ARO, AAV, TDM, FCA, VSG, PRVP, GMAP, LAS, IOAJ and ECPLJ contributed significantly to the drafting of the manuscript. PTCC, JMB and ECPLJ performed critical reviews of the manuscript. ARO, AAV, TDM, FCA, VSG, PRVP, GMAP, LAS, IOAJ, PTCC, JMB and ECPLJ drafted the final version of the manuscript. All authors read and approved the final version of the manuscript.
